# Supplementary material for: The validity and reliability of wearable devices for the measurement of vertical oscillation for running
Source: PLoS One. 2022 Nov 17;17(11):e0277810. doi: 10.1371/journal.pone.0277810 (PMC9671438; doi:10.1371/journal.pone.0277810)
Supplement: Attachment — Submitted filename: Response to Reviewers.docx [file pone.0277810.s002.docx]

Dear Dr Liew,

We would like to thank you and the reviewer for your time reviewing our manuscript. Your feedback has greatly helped us to improve its quality. In response to the comments, the aims and analysis have been restructured to improve the clarity and flow of the manuscript. Please find our responses to each of the points raised below.

Kind regards,

Craig Smith

**Journal requirements**

1. *Please ensure that your manuscript meets PLOS ONE's style requirements, including those for file naming.*

The manuscript has been updated to follow the style requirements.

2. *Please include your full ethics statement in the ‘Methods’ section of your manuscript file. In your statement, please include the full name of the IRB or ethics committee who approved or waived your study, as well as whether or not you obtained informed written or verbal consent. If consent was waived for your study, please include this information in your statement as well.*

The ethics statement is included in the Methods section. Line 81. “All participants gave written informed consent, and the experiment was conducted in accordance with the Declaration of Helsinki. Ethical approval for this research was obtained from the Loughborough University Ethics Review Committee (ERSC22_27).”

3. *Please include captions for your Supporting Information files at the end of your manuscript, and update any in-text citations to match accordingly*.

Supporting information has been added to the end of the manuscript. Line 358.

4. *Thank you for stating the following in the Competing Interests section. Please confirm that this does not alter your adherence to all PLOS ONE policies on sharing data and materials.*

The INCUS NOVA wearable device used in the research article is a license product of INCUS Performance Ltd. CPS, EF, LW, EF, DP, and HL were employees of INCUS Performance Ltd. at the time the research was completed. **This does not alter our adherence to PLOS ONE policies on sharing data and materials.**

**Editor comments**

1. *Please state the hypotheses at the end of the Introduction*

The hypothesis has been included at the end of the Introduction. Line 74.

2. *How did you ensure time synchronisation of all devices? If not, what mechanism was performed to ensure variables from the same time-instance were compared across devices.*

A description of the method for synchronisation of VO measurements across devices has been added to the methods. Line 147.

3. *Each subject had 5 running speeds. Apparently you combined the observations of all subjects and speeds and performed ICC. Have I misinterpreted this? If you have done it, it is incorrect, given that a standard ICC requires individual observations to be independent. Once you have repeated measures, there is variation within each subject that is not accounted for.*

The analysis has been changed from comparing the INCUS NOVA device to the other methods of vertical oscillation measurement to comparing each device to the video analysis.

Now, repeated measures correlations are used to determine the strength of the relationship between the vertical oscillation measurements between the devices and the video analysis across all participants and running intervals.

To test device reliability and validity, we agree with the reviewers comment on ICC and repeated measures, therefore the ICC were re-run but only for a single running interval speed (mid-range speed) per participant. To test reliability, the VO measurements of each device for the mid-range interval were compared between block 1 and 2. For validity, the VO measurements for the two mid-range intervals were averaged and ICC used to compare each device to the video analysis.

4. *please explicitly state the bonferroni correction.*

A description of the Bonferroni correction has been added. Line 156.

5. *provide confidence interval for estimates.*

We are unsure what ‘estimates’ refers to in this comment. Can the Editor please provide more instruction if this comment still applies given the changes made to the analysis.

6. *I am unsure why a comparison between IMU devices are needed. If the aim is to compare which IMU devices is the best, you simply need to compare it with a benchmark. Provide a 95% confidence interval of the error with the benchmark, and that should suffice.*

The analysis has been restructured such that the focus is now on comparisons between each device and the video analysis. Comparisons between the devices are still included in the post-hoc pairwise comparisons and highlight the large differences between some devices. We believe these differences are important as they show that devices cannot necessarily be used interchangeably when measuring vertical oscillation. This point has been made in the discussion.

7. *I think the authors need to think about is the differences in reliability/validity of each device confounded by different locations? Is it a device issue or a location issue, or likely a mixture of both?*

The effect of device location and how vertical oscillation measurement differences between devices is likely a combination of both location and device specific differences has been included in the Discussion. Line 239.

**Reviewer comments**

1. *Line 47: Since you mention that wearable devices provide feedback with "a variety of physiological and biomechanical information", it would be good to include some physiological metrics on top of speed and cadence. OR since your paper is primarily focused on biomechanics, you can exclude physiology and just focus on biomechanics.*

Mention of physiological metrics has been excluded. Line 44.

2. *Line 56: Some examples of visual and auditory feedback would help the reader better understand your claim.*

A description of the type of feedback has been added. Line 53.

3. *Line 60: It should be inertial measurement unit.*

This has been corrected. Line 59.

4. *Lines 75-80: I think the objective statement can flow a little better from the introduction. This paper is clearly aimed towards validating the INCUS NOVA, yet on lines 71-72, you mentioned that the validity and reliability of VO for RDP and Stryd have yet to be validated as well. I recommend either broadening the objective to include the validation of these other devices, or have a smoother transition into the objective that focuses only on INCUS.*

We agree that the objectives of the study could flow better. Therefore, the aims and analysis have been broadened to include validation of all the wearable devices against the video analysis.

5. *Line 105 (and throughout): VMCS is not a standard abbreviation and is confusing. Since you don't use it often, I recommend writing out the full term.*

VMCS has been changed to video analysis throughout.

6. Lines 263-264: I agree that, depending on the device, caution should be taken when interpreting the values. I recommend adding in the discussion what some potential consequences are with overestimating and underestimating VO in terms of performance and injury.

Potential consequences of underestimating or overestimating VO have been added to the Discussion. Line 256.

7. *Figures: All of these figures need to be improved, in my opinion. They are very low quality and the plots don't even have axes.*

The dimension and resolution of the figures have been changed to improve image quality. Axes should now be visible.
