# Supplementary material for: The validity and reliability of wearable devices for the measurement of vertical oscillation for running
Source: PLoS One. 2022 Nov 17;17(11):e0277810. doi: 10.1371/journal.pone.0277810 (PMC9671438; doi:10.1371/journal.pone.0277810)
Supplement: Attachment — Submitted filename: Response to Reviewers.docx [file pone.0277810.s003.docx]

**Response To Reviewers**

**Journal requirements**

1. *Please review your reference list to ensure that it is complete and correct. If you have cited papers that have been retracted, please include the rationale for doing so in the manuscript text, or remove these references and replace them with relevant current references. Any changes to the reference list should be mentioned in the rebuttal letter that accompanies your revised manuscript. If you need to cite a retracted article, indicate the article’s retracted status in the References list and also include a citation and full reference for the retraction notice.*

In text citations have been corrected so that they match the correct reference in the reference list.

**Additional Editor Comments:**

1. *Hypothesis. Can you please state a hypothesis in the form of which method would you think is the best, based on prior research or theory. A directional hypothesis.*

A sentence has been added to the introduction to state that the Footpod device has been shown to be accurate by the manufacturer. This was added to give further reasoning for the directional hypothesis that the devices would be found to be valid. Line 69.

The directional hypothesis has been added. Line 78 – “Based on prior research [14, 15, 16], it is hypothesised that the wearable devices will provide valid and reliable VO measurements when compared to video analysis. However, because of the difference in body locations between devices, it is hypothesised that the VO measurements will differ between devices, with the device in closest proximity to the trunk marker (NOVA) having the most accurate VO measurements when compared to the video analysis measurements.”

2. *Please stated the corrected alpha when stating bonferroni correction - e.g. 0.05/5*

A description of the Bonferroni correction of the p-values by multiplying them by the number of pairwise comparisons made has been added. Line 165 – “the probability values for the paired T-tests were Bonferroni corrected, therefore multiplied by the number of comparisons (i.e. p*10).”

3. *Can you explicitly mention the term time synchronisation ins the paragraph starting with lin 147? E.g. time normalisation was achieved by.....*

The term time synchronisation now starts the paragraph. Line 154.
