# Supplementary material for: The validity and reliability of wearable devices for the measurement of vertical oscillation for running
Source: PLoS One. 2022 Nov 17;17(11):e0277810. doi: 10.1371/journal.pone.0277810 (PMC9671438; doi:10.1371/journal.pone.0277810)
Supplement: Attachment — Submitted filename: Response to Reviewers.docx [file pone.0277810.s004.docx]

**Response To Reviewers**

Dear Dr Liew,

Thank you for your time reviewing our revised manuscript. We have addressed your comment on the Bonferroni correction below and made changes to the manuscript accordingly.

Kind regards,

Craig Smith

**Additional Editor Comments:**

*Before I can accept, there is a mistake in your response. For the Bonferroni correction, you mentioned " the probability values for the paired T-tests were Bonferroni corrected, therefore multiplied by the number of comparisons (i.e. p*10)." If the alpha is 0.05, and it is 0.5, than the correction makes things more lenient. It should be divide. I hope this was a typographical mistake rather than a statistical mistake. Can you clarify?*

The Bonferroni correction was applied to the p-values, multiplying them by the number of comparisons (n=10), therefore making the p-value more conservative, whilst keeping the alpha level at 0.05, e.g. a p-value of 0.04 would be corrected to 0.4 and therefore become non-significant when compared to 0.05. This method of correction is effectively the same as correcting the alpha level by dividing it by the number of comparisons and is how the statistical software package used applies the Bonferroni correction.

To avoid any confusion, the methods and pairwise comparison results have been altered in the manuscript such that instead of the p-values being adjusted, the alpha level is now adjusted for the pairwise comparisons to 0.005.

The methods have been changed to, “*To reduce the likelihood of Type 1 errors when making multiple comparisons,* ***the alpha level for the paired T-tests was Bonferroni corrected, therefore divided by the number of comparisons (i.e. alpha = 0.05/10)****.”* Line 164.

And the pairwise comparison results have been altered. The reported p-values are now uncorrected and compared to the corrected alpha of 0.005. *“Post-hoc pairwise comparisons between devices* ***(Bonferroni corrected alpha level = 0.005)*** *were all significant (t_(14)_ >= 4.32, p < 0.001) other than between HRM and RDP (t_(14)_ = 0.40,* ***p = 0.692****), and NOVA and Footpod (t_(14)_ = 3.16,* ***p = 0.007****).”* Line 181.
